# Clinical Variability and Genotype‐Driven Outcomes in *CHRND*‐Related Congenital Myasthenic Syndrome

**DOI:** 10.1111/ene.70742

**Published:** 2026-08-27

**Authors:** David Muhmann, Göknur Haliloğlu, Maria Antonia Grimalt, Damjan Osredkar, Ana Vesperinas Castro, Silvia Cerezo Corredera, Angela Abicht, Adalet Elçin Yıldız, Johann Böhm, Ulrike Schara‐Schmidt, Anja Troha Gergeli, Berta Estevez‐Arias, Elena Cortes Vicente, Andres Nascimento, Adela Della Marina, Daniel Natera‐de Benito, Andreas Roos

**Affiliations:** ^1^ Department of Neuropediatrics, Developmental Neurology and Social Pediatrics, Centre for Neuromuscular Disorders in Children University Hospital Essen, University of Duisburg‐Essen Essen Germany; ^2^ Division of Pediatric Neurology, Department of Pediatrics Hacettepe University ‐ Faculty of Medicine Ankara Turkey; ^3^ Pediatric Neurology Unit, Pediatric Department Hospital Universitario Son Espases Palma Spain; ^4^ Department of Pediatric Neurology, University Children's Hospital University Medical Centre Ljubljana Ljubljana Slovenia; ^5^ Neuromuscular Diseases Unit, Department of Neurology Hospital de la Santa Creu i Sant Pau Barcelona Spain; ^6^ Department of Medicine Universitat Autònoma de Barcelona Barcelona Spain; ^7^ Biomedical Research Institute Sant Pau (IIB Sant Pau) Barcelona Spain; ^8^ Centro de Investigación Biomédica en Red de Enfermedades Raras (CIBERER), Instituto de Salud Carlos III Madrid Spain; ^9^ Applied Research in Neuromuscular Diseases Institut de Recerca Sant Joan de Déu Barcelona Spain; ^10^ Neuromuscular Unit, Department of Neurology Hospital Sant Joan de Déu Barcelona Spain; ^11^ Medical Genetics Center (MGZ) Munich Munich Germany; ^12^ Department of Radiology Hacettepe University ‐ Faculty of Medicine Ankara Turkey; ^13^ Institut de Génétique et de Biologie Moléculaire et Cellulaire (IGBMC), Inserm U1258, CNRS UMR7104, University of Strasbourg Illkirch France; ^14^ Genomics Unit – NGS, Diagnostic Laboratory Service Hospital Sant Joan de Déu Barcelona Spain; ^15^ Brain and Mind Research Institute Children's Hospital of Eastern Ontario Research Institute Ottawa Ontario Canada; ^16^ Department of Neurology and Heimer Institute for Muscle Research BG‐University Hospital Bergmannsheil, Ruhr University Bochum Bochum Germany

**Keywords:** acetylcholine receptor (AChR), CHRND, congenital myasthenic syndrome (CMS), neonatal hypotonia, ocular symptoms

## Abstract

**Background:**

Congenital myasthenic syndromes (CMS) caused by pathogenic variants in *CHRND*, encoding the δ‐subunit of the nicotinic acetylcholine receptor (AChR), are rare, and data on genotype–phenotype correlations and long‐term outcomes are limited.

**Methods:**

We performed a retrospective, multicenter study of nine patients with genetically confirmed *CHRND*‐related CMS from specialized neuromuscular centers. Clinical, electrophysiological, genetic, and therapeutic data were systematically collected. All diagnoses were established by exome sequencing during routine clinical work‐up.

**Results:**

Eight patients were compound heterozygous and one was homozygous for pathogenic *CHRND* variants, including nonsense, missense, splice‐site variants, and one microdeletion. Disease onset ranged from the neonatal period (*n* = 7) to adolescence (*n* = 2). Three patients were followed longitudinally for 22–43 years. Ocular involvement, particularly ptosis and ophthalmoparesis, was present in all patients. Generalized fatigable weakness was common, whereas bulbar and respiratory involvement occurred in a subset and reflected overall disease severity. Genotypes including a null allele or a homozygous missense variant tended to be associated with more severe phenotypes, while compound heterozygous missense variants were linked to a broader and generally milder spectrum, sometimes limited to ocular symptoms. Long‐term outcomes ranged from minimal symptoms under therapy to severe motor impairment with respiratory insufficiency, highlighting substantial interindividual variability.

**Conclusions:**

This study expands the phenotypic and genotypic spectrum of *CHRND*‐related CMS and underscores the critical role of genotype in determining disease severity. Comprehensive genetic testing, longitudinal phenotyping, and genotype‐informed management are essential for optimal diagnosis and care in this rare disorder.

## Graphical abstract

Clinical variability in CHRND‐related congenital myasthenic syndrome ranges from isolated ocular involvement to severe neonatal‐onset disease with respiratory insufficiency. In a multicenter cohort of nine patients, ocular symptoms represented the core phenotype, while disease severity was influenced by genotype and presumed residual acetylcholine receptor function. Four novel CHRND missense variants were identified, expanding the genetic spectrum and supporting a continuum of disease severity rather than discrete clinical subgroups.

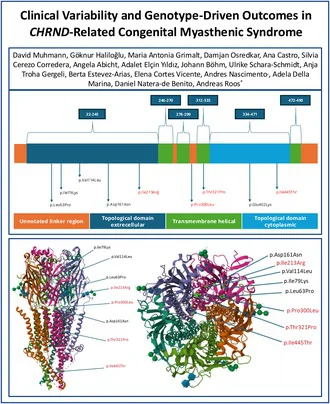

**Abbreviations**
AChR: acetylcholine receptor
ACMG/AMP: American College of Medical Genetics and Genomics/Association for Molecular Pathology
CADD: combined annotation dependent depletion
CMS: congenital myasthenic syndrome(s)
Exome‐seq: exome sequencing
gnomAD: genome aggregation database
LoF: loss‐of‐function
MG: myasthenia gravis
MRI: magnetic resonance imaging
NIV: noninvasive ventilation
NMJ: neuromuscular junction
PVS: pathogenic very strong (ACMG evidence category)
REVEL: rare exome variant ensemble learner
RNS: repetitive nerve stimulation
US: ultrasound

## 1 Introduction

Congenital myasthenic syndromes (CMS) are a heterogenous group of rare inherited disorders, with variable clinical presentation and underlying genetic causes, that are characterized by impaired neuromuscular transmission at the motor endplate. The underlying pathophysiology results from genetic alterations affecting proteins essential for the structure, maintenance, or function of the neuromuscular junction (NMJ) [1]. Over the past two decades, the molecular genetic landscape of CMS has expanded substantially. Most recently, Ohno and colleagues reviewed more than 40 genes implicated in CMS, encompassing defects of presynaptic, synaptic, and postsynaptic components of the neuromuscular junction [2]. The growing list of causative genetic defects moreover points towards a fourth category comprising ubiquitously expressed proteins involved in general cellular pathways (e.g., glycosylation) that secondarily impair neuromuscular transmission in addition to the more clearly defined as presynaptic, synaptic, and postsynaptic forms [3, 4]. Striking examples are genes such as *PTPN11* and *PURA* underscoring this increasing genetic, clinical and mechanistic heterogeneity of these disorders [2].

Postsynaptic CMS represents a major subgroup, with postsynaptic acetylcholine receptor (AChR) subunit genes collectively accounting for a substantial fraction of cases. The *CHRND* gene encodes the δ‐subunit of the nicotinic AChR, an essential component of the pentameric receptor complex at the neuromuscular junction. Loss‐of‐function variants in *CHRND* reduce or abolish functional AChR expression at the postsynaptic membrane, resulting in an AChR‐deficiency‐type CMS [5]. *CHRND*‐associated CMS was first described in 2001 by Brownlow and colleagues reporting on a child with neonatal onset of arthrogryposis multiplex congenita based on biallelic *CHRND* variants [6]. However, while *CHRND* is one of the causative genes for CMS, its contribution to overall CMS cases is much smaller than the major subtypes like *CHRNE, RAPSN, COLQ* and *DOK7* and clinical long‐term observations are lacking. Most published genetic surveys report *CHRND* as a rare cause of CMS (e.g., [7]), thus limiting the current understanding of genotype–phenotype correlations which would address the question of whether such correlations are meaningful at all. Preclinical studies have shown that pathogenic *CHRND* variants impair co‐clustering of the AChR with rapsyn, resulting in a marked reduction of AChR density at the NMJ [8].

Given the rarity of *CHRND*‐associated CMS and the expanding spectrum of pathogenic variants, detailed clinical characterization of patients in conjunction with genetic findings is essential for accurate diagnosis, prognostic assessment, and therapeutic decision‐making. In this study, we report on nine patients with *CHRND*‐related CMS, highlighting correlations between genotypes and clinical severities to improve the current understanding of this rare CMS subtype.

## 2 Materials and Methods

This retrospective, multicenter study included patients recruited from the Department of Pediatric Neurology of University Hospital Essen (Germany), from the Division of Pediatric Neurology at Hacettepe University Faculty of Medicine Ankara (Turkey), from the Neuromuscular Unit of Hospital Sant Joan de Déu, Barcelona (Spain), from the Department of Neurology at Hospital de la Santa Creu i Sant Pau Barcelona (Spain), and from the Department of Child, Adolescent and Developmental Neurology of University Children's Hospital Ljubljana (Slovenia). All patients were evaluated by experienced neuromuscular specialists. Long‐term clinical follow‐up under therapeutic intervention was conducted for three patients at the respective clinical centers in Barcelona, Essen, and Ljubljana. All clinical data were collected retrospectively during routine clinical assessments. Genetic diagnoses were established via exome sequencing as part of the routine diagnostic work‐up.

For clinical contextualization and comparison, our patients were compared with previously reported cases, including the prior clinical descriptions of the three patients for whom we present longitudinal follow‐up data [8, 9, 10, 11].

## 3 Results

### 3.1 Clinical Findings

**FIGURE 1:**
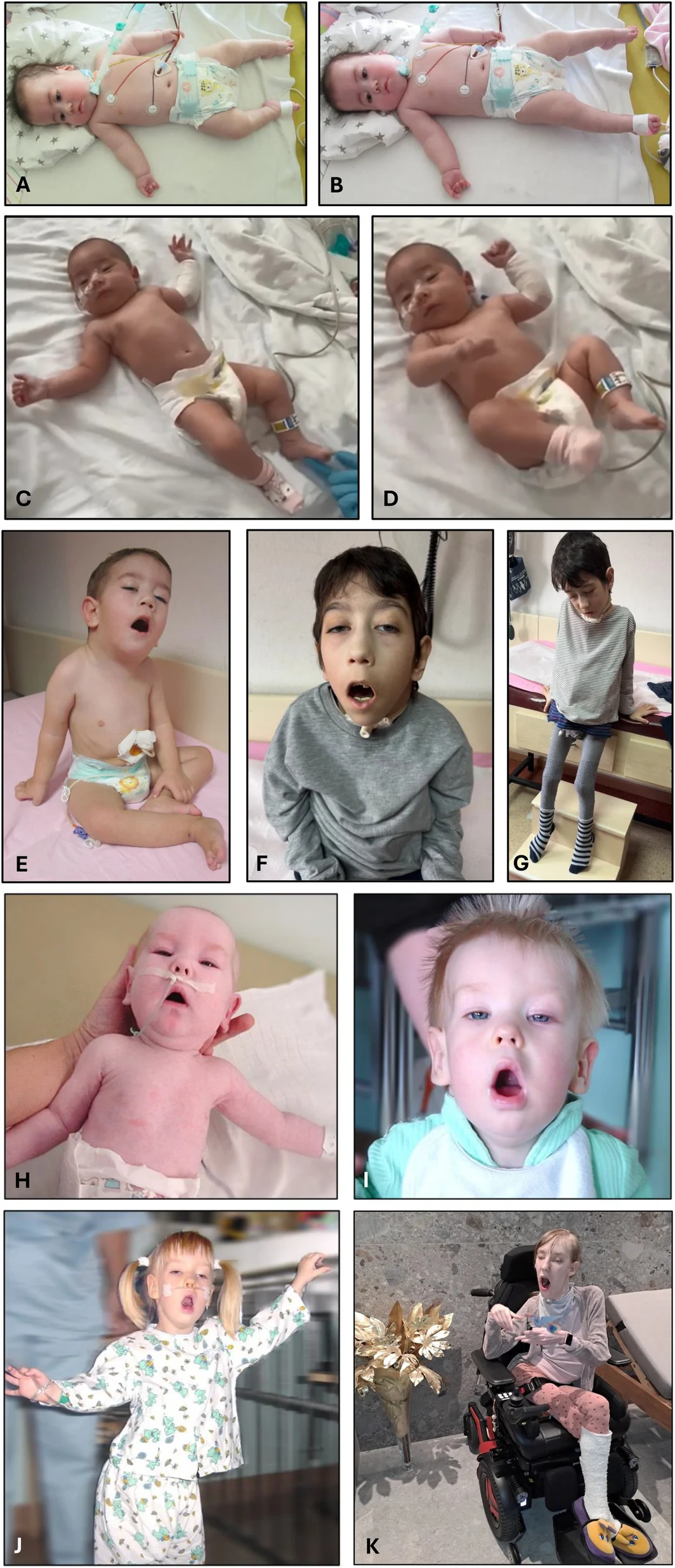
Clinical features of patients with *CHRND*‐associated CMS (neonatal‐onset): (A) Patient 1 at 8 months of age presenting with facial hypomimia and ophthalmoparesis (not shown). Due to severe respiratory insufficiency, the patient required tracheostomy and continuous mechanical ventilation. A gastrostomy was performed because of bulbar dysfunction. (B) Patient 1 exhibited severe generalized muscle weakness with pes equinovarus and was able to lift her lower extremities only minimally against gravity. (C) Patient 5 at 3 months of age presenting with facial hypomimia, severe bilateral ptosis, and nasogastric tube placement due to bulbar dysfunction. (D) Despite generalized weakness, Patient 5 was able to elevate the extremities against gravity. (E) Patient 8 at 27 months of age presenting with facial hypomimia, severe bilateral ptosis, and nasogastric tube placement due to bulbar dysfunction. (F) At 3 years of age, Patient 8 underwent tracheostomy because of recurrent respiratory crises. (G) Patient 8, at the age of 14 years and 2 months, achieved motor milestones, including independent sitting by 8 months of age and walking by 4 years of age, but showed marked muscular hypotrophy of the extremities. (H) Patient 9 at the age of 4 months showing signs of generalized hypotonia, ptosis, tent‐shaped open mouth, and the need for nasogastric tube due to poor feeding. (I) Patient 9 showing dysmorphic features, hypotonia, and ptosis at the age of 1 year. (J) Patient 9 at the age of 9 years presenting with signs of hypotonia, ptosis and scoliosis, while she was still able to walk with support. (K) Patient 9 at the age of 22 years with signs of severe hypotonia, scoliosis, contractures, dystrophia, and wheelchair dependence.

All clinical and demographic findings are summarized in Table 1. Representative clinical photographs of selected patients are presented in Figure 1, and the corresponding pedigrees are provided in Figure S1. The cohort consisted of five females and four males, ranging in age at last follow‐up from 9 months to 43 years. Symptom onset was neonatal in seven patients and occurred during adolescence in two. All patients exhibited ocular involvement, with ptosis in all cases and ophthalmoparesis or ophthalmoplegia in six (66.7%). Five patients had strabismus. Generalized fatigable weakness of variable severity with bulbar involvement and swallowing difficulties was observed in seven individuals (77.8%). Among the neonatal‐onset individuals, four (57%; Patients 1, 5, 8 and 9) presented with severe disease, including marked bulbar and respiratory symptoms, and required tracheostomy and/or gastrostomy. The remaining three neonatal‐onset individuals (43%; Patients 2, 3, 4) showed ocular symptoms with milder generalized weakness that improved over time, presenting predominantly ocular manifestations with mild proximal weakness and myopathic facies at last follow‐up. The two adolescent‐onset individuals (Patients 6, 7) had isolated ocular involvement without generalized weakness or relevant respiratory or gastrointestinal issues.

**TABLE 1:** Clinical characteristics of patients with *CHRND*‐associated CMS.

Patient	1	2	3	4	5	6 (Sibling of 7)	7 (Sibling of 6)	8	9
Sex	F	M	F	F	F	M	M	M	F
Previosly reported	/	[8, 9]	/	[11]	/	/	/	/	[10]
Current age	3 years	27 years	13 years	43 years	9 months	18 years	18 years	14 years	27 years
Age at onset	Neonatal	Neonatal	Neonatal	Neonatal	Neonatal	Adolescence	Adolescence	Neonatal	Neonatal
Age at cliinical diagnosis	0 years	8 years	1 year	0 years	0 years	16 years	17 years	2 years	0 years
Initial manifestation	Prematurity (35 GW); neonatal respiratory failure; generalized hypotonia/weakness; talipes equinovarus, lagophthalmos, bulbar dysfunction; ophthalmoplegia; high arched palate	Feeding difficulties; weak cry; facial weakness; hypersomnolence	Ptosis; recurrent infections; feeding difficulties; mild generalized hypotonia; facial weakness	Ptosis; recurrent infections; hypotonia; feeding difficulties	Generalized hypotonia/weakness; bulbar dysfunction	Ophthalmoparesis	Ophthalmoparesis	Prenatal hypokinesia; generalized hypotonia; neonatal respiratory failure; seizures (normal EEG); bulbar dysfunction; high‐arched palate	Prenatal hypokinesia; polyhydramnion; neonatal respiratory failure; generalized hypotonia, ptosis, feeding difficulties
Core phenotype	Generalized hypotonia/weakness; facial weakness; ocular involvement (ptosis, ophthalmoplegia, lagophthalmos)	Infection induced fatigability, ocular involvement (intermittent ptosis/diplopia/strabismus); joint contractures	Isolated ocular phenotype (stress or fatigue‐induced ptosis, strabismus; diplopia)	Moderate proximal weakness; facial weakness; fatigability; ocular involvement (ptosis/strabismus/intermittent diplopia)	Generalized hypotonia/weakness, facial weakness; ocular involvement (ptosis/ophthalmoparesis)	Isolated ocular phenotype (ophthalmoparesis, fluctuating ptosis, strabismus)	Isolated ocular phenotype (ophthalmoparesis, fluctuating ptosis, strabismus)	Generalized hypotonia; axial/proximal weakness; facial hypomimia; generalized muscle atrophy; ocular involvement (ptosis/ophthalmoplegia)	Generalized hypotonia/weakness; facial hypomimia, muscle; hypotrophy; areflexia; ocular involvement (ptosis/ophthalmoplegia)
Respiratory/gastrointestinal involvement	Tracheostomy with continuous ventilation; gastrostomy dependence	None	None	None (FVC 81%)	Dysphagia; gastrostomy dependence; aspiration pneumonia at 8 months	None	None	Tracheostomy at the age of 3 years, NIV BiPap at age 7–8; failure to thrive; gastrostomy dependence	Tracheostomy dependence; ventilator dependence (~16 h/day); dysphagia; gastrostomy dependence
Orthopdedic symptoms	Talipes equinovarus; claw hands; joint contractures, scoliosis	Small joint and knee contractures	None	None	None	None	None	Mild thoracic scoliosis; lumbar lordosis; knee flexion contractures	Talipes equinovarus; talocrural joint contractures; kyphoscoliosis; dolihocephalia
Functional status at last follow‐up	Nonambulatory; non‐sitting	Preserved ambulation	Isolated ocular phenotype	Wheelchair use for daily activities	Independent sitting	No motor impairment	No motor impairment	Waddling gait; unable to run/jump/climb stairs	Nonambulatory; non‐sitting
Disease course/outcome	Severe stable phenotype	Stable course	Stable course	Stable course	Stable course with mild improvement	Stable ocular phenotype	Stable ocular phenotype	Severe stable course; cognitive impairment	Severe slowly progressive disease course
Repetitive nerve stimulation	Pathological decrement	Pathological decrement	Pathological decrement	Pathological decrement	Pathological decrement not identified	Not performed	Not performed	Not performed	Pathological decrement
Therapy response	Sal(+), PS(+), 3,4‐DAP (−)	PS(+)	PS(+)	PS(+), 3,4‐DAP(+), Ephedrine(+), Sal(+), fluoxetine (worsening)	PS (+)	PS(−)	PS(−)	PS (+) and 3,4 DAP(+) Sal (−)	P (+); 3,4‐DAP (+)
Ongoing treatment	Sal: 7 mg/day PS: 5.7 mg/kg/day	PS: 270 mg/day	PS: 5.2 mg/kg/day	Sal: 8 mg/day PS: 480 mg/day, 3,4‐DAP: 70 mg/day	PS: (8 mg/kg/day)	No treatment (PS 5 mg/kg/day for 1 year no change)	No treatment (PS 5 mg/kg/day for 1 year no change)	Sal: 2.4 mg/day PS: 7.5 mg/kg/day Amifamprinine (10 mg/day)	PS:13.8 mg/kg/day 3,4‐DAP:1.4 mg/kg/day
MRI/imaging findings	Brain MRI: lower limit of normal maturation	Not performed	Not performed	Muscle MRI: Bilateral soleus atrophy Grade II, medial gastrocnemius focal Grade II–III	Brain MRI: normal	Not performed	Not performed	Brain MRI (as newborn): normal Muscle US: Increased echogenicity and mild atrophy of sartorius, medial and lateral heads of gastrocnemius	Not performed

*Note:* The table summarizes demographic data, age at symptom onset and diagnosis, initial clinical presentation, neuromuscular, ocular, respiratory, gastrointestinal, and orthopedic involvement, findings at last follow‐up, results of repetitive nerve stimulation, therapeutic responses, ongoing treatments, and available neuroimaging or muscle imaging findings for nine affected patients. Disease severity ranged from severe neonatal‐onset generalized weakness with bulbar and respiratory involvement to milder, predominantly ocular phenotypes with adolescent onset.

Abbreviations: 3,4‐DAP, 3,4‐diaminopyridine (amifampridine); FVC, forced vital capacity; MRI, magnetic resonance imaging; NIV, noninvasive ventilation; PS, pyridostigmine; Sal, salbutamol; US, ultrasound.

Repetitive nerve stimulation was performed in six patients (66.7%) and revealed a pathological decrement in five (83%), consistent with impaired neuromuscular transmission. Brain MRI in two patients (Patient 1 and 8) showed no structural abnormalities. Muscle MRI in another patient (Patient 4) revealed selective atrophy of the bilateral soleus and medial gastrocnemius muscles (data not shown). Muscle ultrasound was performed in Patient 8 at 14 years old and demonstrated, in addition to selective sartorius involvement, abnormalities of the gastrocnemius and paraspinal muscles (Figure S2). These findings likely reflect long‐standing disease‐related changes.

All patients were treated with pyridostigmine and clinical improvement was observed in seven (77.8%). Three patients were furthermore treated with salbutamol or ephedrine, with additional improvement in two (66.7%). Four patients underwent additional treatment with 3,4‐diaminopyridine which resulted in clinical benefit in three (75%). The two adolescent‐onset patients with isolated ocular involvement did not show a meaningful response with pyridostigmine despite prolonged treatment. Of note, both patients were tested negative for anti‐AChR and anti‐MuSK antibodies, supporting the diagnosis of congenital rather than autoimmune myasthenia. Long‐term follow‐up of three patients (Patients 2, 4, and 9) over a period of 22 to 43 years (Table 1) revealed a broad clinical outcome under therapy. This ranged from very mild muscular symptoms, characterized by infection‐triggered exercise intolerance combined with mild ocular involvement in Patient 2, to severe generalized muscular impairment with inability to sit and dependence on mechanical ventilation via tracheostomy in Patient 9 (Table 1).

In six patients, older than 13 years at their last follow‐up, cognitive development appeared normal; all attended or had previously attended mainstream schools. In two neonatally affected patients (Patient 1 and 9), standardized neuropsychological testing was not feasible due to profound motor impairment, precluding reliable assessment of intellectual functioning; however, Patient 1 demonstrated appropriate responsiveness and nonverbal interaction despite tracheostomy dependence. One additional neonatally affected patient (Patient 8) exhibited significant intellectual disability accompanied by behavioral abnormalities and absent language at the age of 14 years. Formal neuropsychological testing was not performed.

### 3.2 Molecular Genetic Findings

All patients had biallelic pathogenic or likely pathogenic variants in *CHRND* (NM_000751.3) (Table 2). Four individuals carried exclusively missense variants: three were compound heterozygous (Patients 4, 6, and 7), while one was homozygous for a missense variant (Patient 8). The remaining cases presented a combination of one missense and one nonsense variant (Patients 1 and 5), or one missense and one splice‐site variant (Patients 3 and 9), which were predicted to be truncating (null alleles). One patient presented with a combination of a missense variant and a microdeletion spanning exon 8 to intron 9 (Patient 2; chr2:232531431‐2:232533648, GRCh38.p14). Four missense variants, p.Ile213Arg, p.Pro300Leu, p.Thr321Pro and p.Ile445Thr, have not previously been reported (Table 2).

**TABLE 2:** Molecular genetic findings in patients with *CHRND*‐associated CMS.

Patient	Variant (cDNA; Protein)	Variant type	Zygosity	ΔΔG	CADD/REVEL	ACMG evidence codes	Classification	Published
1	c.234G>A; p.Trp78Ter	Null allele	Compound heterozygous			PVS1, PM2, PM3	Pathogenic	[12]
	c.961A>C; p.Thr321Pro	Missense		−0.13	28.5/0.7	PM2, PP3	Variant of uncertain significance	—
2	c.1204G>A; p.Glu402Lys	Missense	Compound heterozygous	0.74	25.2/0.74	PM2, PP3, PM3, PS3	Pathogenic	[8, 13]
	Microdeletion exon 8–intron 9	Microaberration				PVS1, PM2	Likely pathogenic	[8]
3	c.1204G>A; p.Glu402Lys	Missense	Compound heterozygous	0.74	25.2/0.74	PM2, PP3, PM3, PS3	Pathogenic	[8, 13]
	c.821‐2A>C	Splice‐site → null allele				PVS1, PM2, PM3	Pathogenic	[10, 14]
4	c.188T>C; p.Leu63Pro	Missense	Compound heterozygous	3.48	27.8/0.9	PM2, PP3	Variant of uncertain significance	[11]
	c.236T>A; p.Ile79Lys	Missense		2.61	26.3/0.83	PM2, PP3, PS3	Likely pathogenic	[11]
	c.340G>C; p.Val114Leu	Missense		0.5	25.6/0.63	PM2	Variant of uncertain significance	[11]
5	c.1006C>T; p.Arg336Ter	Null allele	Compound heterozygous			PVS1, PM2	Likely pathogenic	[15]
	c.1334T>C; p.Ile445Thr	Missense		1.32	25.3/0.97	PM2, PP3, PM3	Likely pathogenic	—
6	c.236T>A; p.Ile79Lys	Missense	Compound heterozygous	2.61	26.3/0.83	PM2, PP3, PS3	Likely pathogenic	[11]
	c.899C>T; p.Pro300Leu	Missense		0.19	24.7/0.94	PM2, PP3, PM3	Likely pathogenic	—
7	c.236T>A; p.Ile79Lys	Missense	Compound heterozygous	2.61	26.3/0.83	PM2, PP3, PS3	Likely pathogenic	[11]
	c.899C>T; p.Pro300Leu	Missense		0.19	24.7/0.94	PM2, PP3, PM3	Likely pathogenic	—
8	c.638T>G; p.Ile213Arg	Missense	Homozygous	2.65	25.5/0.96	PM2, PP3, PM3	Variant of uncertain significance	—
9	c.481G>A; p.Asp161Asn	Missense	Compound heterozygous	0.82	31/0.88	PM2, PP3	Variant of uncertain significance	[10]
	c.821‐2A>C	Splice‐site → null allele				PVS1, PM2, PM3	Pathogenic	[10, 14]

*Note:* The table summarizes *CHRND* variants identified in nine affected patients, including cDNA and protein nomenclature, variant type, zygosity, applied ACMG/AMP evidence codes, CADD/REVEL, ΔΔG and final variant classification. Previously published variants and their corresponding references are indicated where applicable. Most patients carried compound heterozygous variants, comprising missense, nonsense, splice‐site, and structural variants, consistent with autosomal recessive inheritance. Variant interpretation was performed according to ACMG guidelines.

Abbreviations: PM2, absent or rare in population databases; PM3, detected in trans with a pathogenic variant in a recessive disorder; PP1, segregation with disease in affected family members; PP3, multiple lines of computational evidence supporting a deleterious effect; PS3, well‐established functional studies supportive of damaging effect; PVS1, predicted loss‐of‐function variant.

According to the ACMG/AMP guidelines [16], the nonsense variants (p.Trp78Ter and p.Arg336Ter) were classified as pathogenic, as they are predicted to result in loss‐of‐function in a gene with a well‐established disease mechanism (PVS1). Based on *in silico* splicing analyses, the splice‐site variant c.821‐2A>C is predicted to abolish the canonical acceptor splice‐site at the intron 7‐exon 8 junction (SpliceAI score = 0.97, position = +2 bp), and to activate a cryptic acceptor site within intron 7 (SpliceAI score = 0.36, position = −123 bp). This aberrant splicing is expected to result in partial retention of intron 7, leading to a frameshift and the introduction of a premature termination codon, classified as pathogenic under the PVS1 criterion. The microdeletion affecting exon 8 and intron 9 (in combination with a missense variant) has already been reported in the previous publication of Patient 2 by Müller and colleagues [8]. However, experimental evidence supporting a true null allele is lacking. A more precise deletion analysis shows the breakpoint is located near the distal end of exon 8, suggesting exon 8 is not fully deleted. Additionally, a putative splice donor site is present in intron 9 downstream of the breakpoint. Thus, this microdeletion may be hypomorphic, allowing residual transcript production and the generation of an alternatively spliced transcript.

All identified *CHRND* missense variants, including p.Leu63Pro (CADD = 27.8, REVEL 0.9), p.Ile79Lys (CADD = 26.3, REVEL 0.83), p.Val114Leu (CADD = 25.6, REVEL 0.63), p.Asp161Asn (CADD = 31, REVEL 0.88), p.Ile213Arg (CADD = 25.5, REVEL 0.96), p.Thr321Pro (CADD = 28.5, REVEL 0.7), p.Pro300Leu (CADD = 24.7, REVEL 0.94), p.Glu402Lys (CADD = 25.2, REVEL 0.74) and p.Ile445Thr (CADD = 25.3, REVEL 0.97) exhibited consistently high CADD scores (> 20), supporting deleterious effects on protein function (PP3). As siblings, Patients 6 and 7 harbored identical compound heterozygous missense variants (c.236T>A; p.Ile79Lys and c.899C>T; p.Pro300Leu). Several variants have previously been reported in association with CHRND‐related CMS, including p.Asp161Asn [10], and the three missense variants identified in Patient 4 [11], providing additional evidence supporting their pathogenicity (Table 2).

Furthermore, all variants were absent from the gnomAD (v4.1.0) or observed at extremely low heterozygous allele frequencies, consistent with the PM2 criterion. Comprehensive reference searches and clinical variant databases (e.g., ClinVar, GenCC) do not list these variants with appreciable allele frequency, which usually correlates with absence or an allele frequency < 0.00001 in gnomAD. Taken together, these genetic findings support *CHRND* as the causative gene in all patients included in this cohort.

Segregation analyses confirmed autosomal recessive inheritance in all families. Two of the novel variants p.Thr321Pro and p.Ile445Thr were identified in trans with pathogenic nonsense variants, providing additional evidence for pathogenicity. Notably, the affected residues of the novel variants (Ile213, Pro300, Thr321, and Ile445) are highly conserved across species.

**FIGURE 2:**
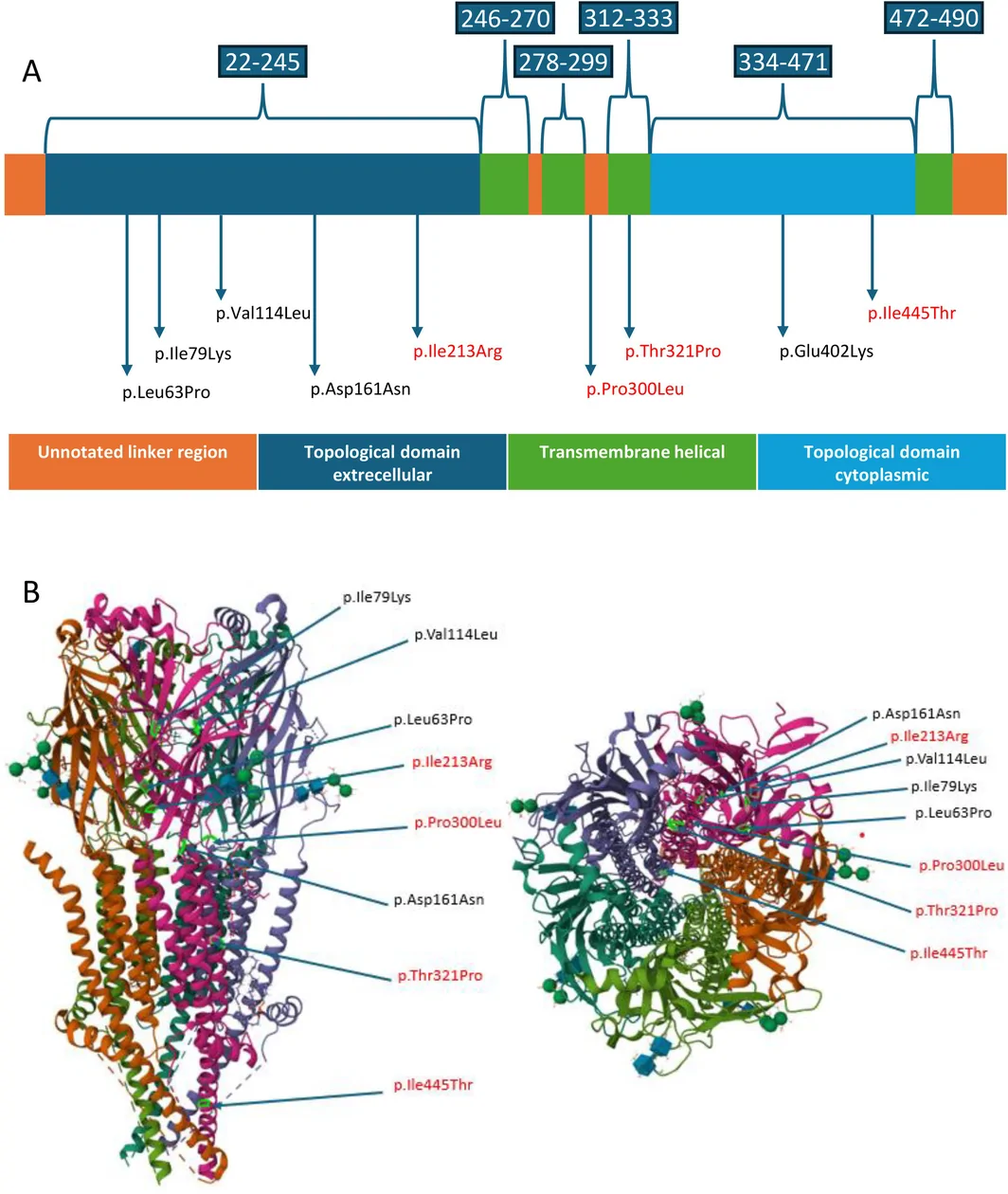
Structural localisation of CHRND missense variants within the human muscle acetylcholine receptor: (A) Schematic representation of the CHRND δ‐subunit showing extracellular, transmembrane helical, and cytoplasmic domains together with the localisation of identified missense variants. Novel variants identified in the present cohort are highlighted in red. (B) Structural mapping of CHRND missense variants onto the human muscle acetylcholine receptor cryo‐EM structure (PDB: 9DMH) shown in side and top views. Of note: The p.Glu402Lys variant could not be reliably mapped onto the available cryo‐EM structure because the corresponding region was incompletely resolved in the structural model.

Structural mapping using Alpha Fold models and the human muscle AChR cryo‐EM structure (PDB: 9DMH) showed that variants are located in distinct regions of the δ‐subunit, including extracellular, transmembrane, and cytoplasmic domains, with some located near subunit interfaces (Figure 2 and Figure S3).

**FIGURE 3:**
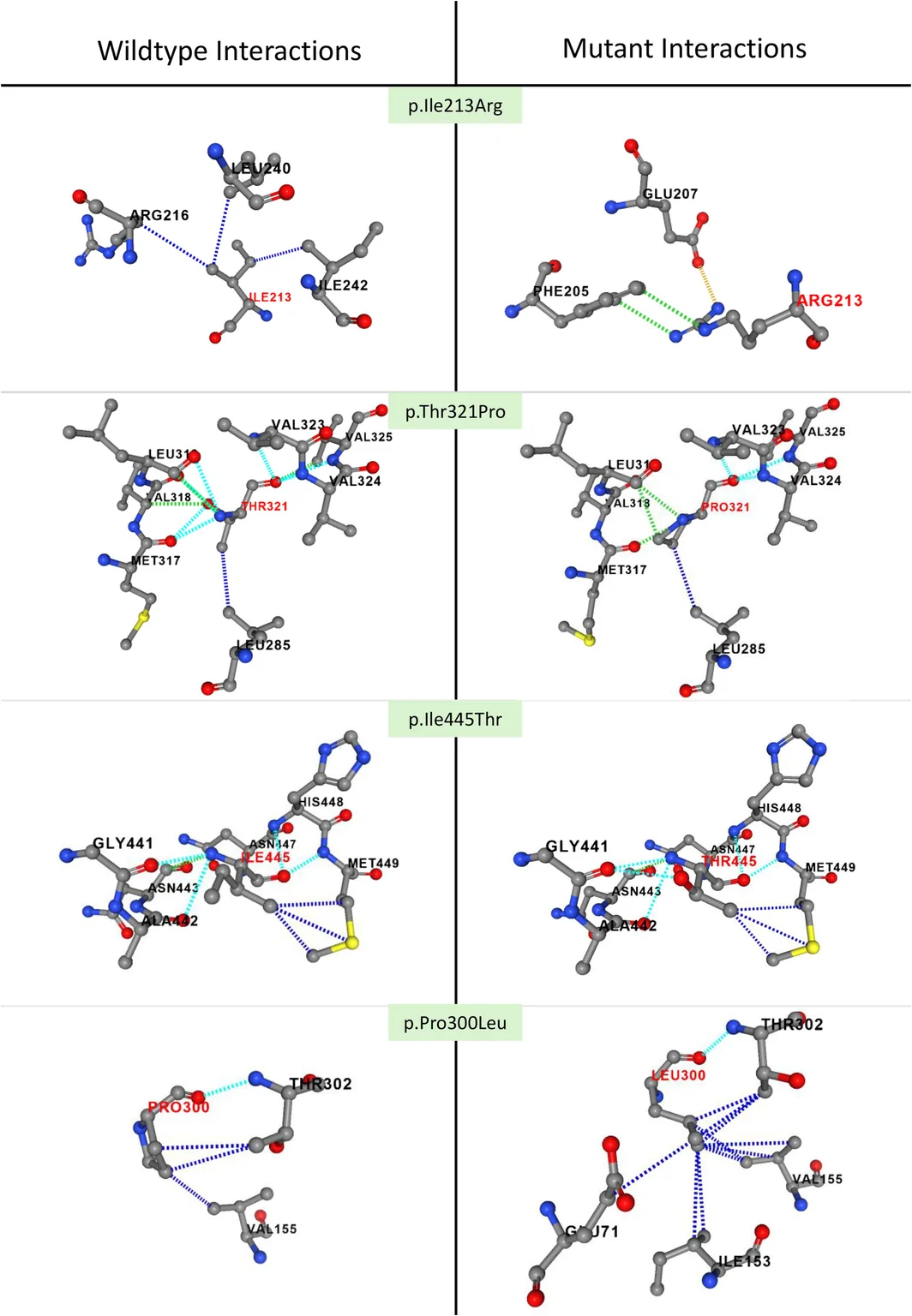
Comparison of the local residue interaction networks between wild‐type and mutant CHRND residues for p.Ile213Arg, p.Thr321Pro, p.Ile445Thr, and p.Pro300Leu based on PremPS [17]: For each variant, the left panel shows the wild‐type residue interactions and the right panel the corresponding mutant residue interactions. The analyses demonstrate variant‐specific alterations in local interaction patterns and residue environments, supporting heterogeneous structural consequences of CHRND missense variants. Interaction types are indicated as follows: Blue dashed lines, hydrophobic interactions; turquoise dashed lines, polar interactions; yellow dashed lines, ionic interactions; green dashed lines, van der Waals (VDW) interactions. Structural modeling provides supportive structural evidence only and does not establish the precise functional consequences of the variants.

Protein stability predictions using PremPS [17] revealed variant‐related differences in ΔΔG values and local interaction networks, highlighting heterogeneous structural perturbations associated with the different CHRND missense variants (Table 2; Figure 3).

## 4 Discussion

Pathogenic variants in *CHRND* are a rare cause of CMS [5]. While dominant CHRND‐related slow‐channel CMS has been reported (MIM: 616321), such cases are rather uncommon and are not part of the present cohort, which focuses on biallelic *CHRND* variants (MIM: 616323). Of these, eight patients were compound heterozygous and one homozygous, consistent with autosomal recessive inheritance. Three patients (Patients 2, 4, and 9) had been previously reported [8, 9, 10, 11]. Identified variants included nonsense, missense, and splice‐site changes as well as a microdeletion affecting critical regions of the δ‐subunit. The 2.2‐kb microdeletion encompassing exon 8 to intron 9 of Patient 2 constitutes the only microaberration reported to date in association with a *CHRND*‐related CMS [8]. Four missense variants, p.Ile213Arg, p.Pro300Leu, p.Thr321Pro, and p.Ile445Thr, have thus far not been reported in the literature and thus expand the current genetic landscape of *CHRND*‐related CMS.

Across the cohort, ocular involvement with ptosis and ophthalmoparesis/plegia represents the core manifestation of *CHRND*‐related CMS (Tables 1 and 3). Additional features, including fatigable weakness, bulbar dysfunction, and respiratory insufficiency, varied and were largely determined by the underlying genotype: Individuals carrying biallelic missense variants tended to present milder phenotypes dominated by ocular symptoms, although variability was observed within this group. Notably, neonatal onset was not invariably associated with severe disease, as some patients with early presentation showed predominantly ocular involvement, underscoring that age at onset alone is not a reliable predictor of severity. Patients harboring one null allele in combination with a missense variant constituted the most severely affected end of the spectrum, presenting with profound neonatal hypotonia, generalized fatigable weakness, bulbar involvement, and early respiratory compromise requiring supportive measures. However, this observation should be interpreted with caution. While such genotypes are compatible with reduced residual AChR activity due to combined quantitative and qualitative impairment, phenotypic variability exists, and variant class alone does not fully determine clinical severity. This is underlined by the presence of two patients of our cohort with predicted out‐of‐frame splice‐site variants or microdeletions and comparatively milder phenotypes. Conversely, missense variants may cause severe disease depending on their functional effects [1]. Therefore, the concept of a genotype‐driven severity gradient should be regarded as a general tendency rather than a strict rule. All missense variants showed high CADD/Revel scores, consistent with a predicted deleterious impact on protein function.

**TABLE 3:** Previously reported patients with *CHRND*‐associated CMS and fetal akinesia/multiple pterygium spectrum disorders.

References	[6]	[18]			[12]							[19]	[20]		[15]
Sex	♀	♂	♂	♀	♂ (fetus)	♂ (fetus)	♀ (fetus)	♀ (fetus)	♂ (fetus)	♂ (fetus)	♂ (fetus)	♂	♀	♀	♂ (fetus)
Genetic variant(s)	c.756_757ins & p.Glu59Lys	p.Pro250Gln			c.234G>A; p.Trp78Ter		c.283 T>C; p.Phe95Leu & c.1390C>T; p.Arg464Ter					c.59G>A; p.Trp20Ter & c.423G>C; p.Gly141Arg	c.730C>T; p.Arg244Cys & c.1304 T>C; p.Leu435Pro		c.1006C>T; p.Arg336Ter & c.973_975delGTG; p.Val 325del
Zygosity	Comp. Het.	Hom.			Hom.		Comp. Het.					Comp. Het.	Comp. Het.		Comp. Het.
Functional mechanism	Null allele & missense	Missense			Null allele; Null allele & missense							Null allele & missense	Missense		Null allele & In‐frame deletion of one amino acid
Age at onset	Neonatal	Neonatal			Prenatal							8 years	Neonatal		Prenatal (first trimester)
Age at diagnosis	4 months	NR			NR							NR	NR		Prenatal
Initial symptoms	Arthrogryposis; neonatal hypoxia; feeding difficulty; floppy infant; weak cry	Poor suck/cry; ventilatory support	Poor respiratory effort at birth		Growth retardation; intrauterine edema; decreasedmovements; contractures	Growth retardation; intrauterine edema; cystic hygroma; decreased movements; contractures; reduced muscle bulk/hypoplasia; scoliosis; Pterygia; Pectus excavatum; broad rips, claviculae, and os metatarsale	Intrauterine edema; depressed nasal bridge; low‐set ears; generalized edema; big atrial septal defect; lung hypoplasia; hydrothorax; ascites	Cystic hygroma; joint contractures; faciocranial dysmorphism; hypertelorism; depressed nasal bridge; micrognathia; low‐set ears; contractures; Pterygia; rocker bottom feet; lung hypoplasia; hydrothorax; pericardial effusion; shortened ribs	Cystic hygroma; decreased movements; contractures, Pterygia	Growth retardation; edema/cystic hygroma; joint contractures; faciocranial dysmorphism; downslanting palpebral fissures; hypertelorism; depressed nasal bridge; micrognathia; low‐set ears; contractures; pterygia	Growth retardation; edema; cystic hygroma; joint contractures; faciocranial dysmorphism; downslanting palpebral fissures; hypertelorism; depressed nasal bridge; micrognathia; low‐set ears; contractures; pterygia	Progressive muscle weakness; chewing difficulty	Generalized hypotonia; bulbar failure; weak cry; severe ptosis; ophthalmoparesis, respiratory failure		Increased nuchal translucency, absent nasal bone, edema, reduced/absent limb movement, contractres, post termination multiple pterygia/contractures and craniofacial abnormalities
Neuromuscular symptoms	Fatigable limb weakness	Delayed motor development, facial diplegia, fatigue on exertion, weak lingual, masticatory and neck flexor muscles			NR							Facial weakness, fatigability	Severe generalized weakness and hypotonia		Fetal akinesia sequency/multiple pterygia phenotype
Ocular symptoms	Ptosis; opthalmoplegia	Ptosis, ophthalmoplegia			NR							NR	Severe ptosis and opthalmoplegia		NR
Respiratory symptoms	Severe neonatal respiratory distress	NR		Restrictive ventilatory defect after the age of 18	NR							Postoperative respiratory insufficiency requiring mechanical ventilation	Acute neonatal respiratory distress requiring invasive ventilation		NR
Gastrointestinal symtpoms	Dysphagia	NR			NR							Chewing difficulty	Severe sucking and swallowing impairment		NR
Orthopedic symptoms	Arthrogryposis multiplex congenita	Thoracolumbar scoliosis			NR							Scoliosis	NR		NR
Nerve conduction velocity	Increased jitter with blocking	Decremental response			NR							Decremental response	NR		NR
Therapy response	PS+	PS (partial) and 3,4,‐DAP (+)			NR							PS (partial)	Neostigmine (largely ineffective), partial response to Sal		NR
Ongoing treatment	NR	NR			NR							NR	NR		NR
MRI/imaging findings	NR	cMRI: symmetric bithalamic foci of increased T2 and decreased T1 signal	NR		NR							NR	NR		Prenatal ultrasound abnormalities

*Note:* The table summarizes published cases carrying pathogenic or likely pathogenic *CHRND* variants, including sex, genetic variants and zygosity, predicted functional mechanisms, age at onset and diagnosis, initial clinical presentation, neuromuscular, ocular, respiratory, gastrointestinal, and orthopedic manifestations, electrophysiological findings, treatment response, ongoing therapy, and available neuroimaging or prenatal ultrasound findings. Phenotypes range from prenatal lethal multiple pterygium syndrome with fetal akinesia to postnatal congenital myasthenic syndrome with variable severity. Legacy variant nomenclature was converted to current HGVS‐compliant annotation where possible.

Abbreviations: 3,4‐DAP, 3,4‐diaminopyridine; cMRI, cerebral MRI; Comp. Het., compound heterozygous; Hom., homozygous; MRI, magnetic resonance imaging; NR, not reported; PS, pyridostigmine; Sal, salbutamol.

To investigate potential molecular mechanisms, we conducted an in silico structural analysis in which the missense variants were mapped onto the available human muscle AChR cryo‐EM structure (PDB: 9DMH). Several variants (p.Thr321Pro, p.Ile445Thr, p.Pro300Leu and p.Val114Leu) are located near to inter‐subunit interfaces, suggesting possible effects on subunit interactions. Others such as p.Leu63Pro, p.Ile79Lys, p.Ile213Arg and p.Asp161Asn are not directly positioned at these interfaces and may predominantly affect local protein folding or stability (Figure 2 and Figure S3). Consistent with this observation, stability predictions using PremPS demonstrated variable effects across the cohort, with some variants (e.g., p.Leu63Pro, p.Ile79Lys and p.Ile213) showing positive ΔΔG values consistent with predicted protein destabilization and others showing only minor changes (e.g., p.Val114). For the novel variants, p.Ile213Arg and p.Ile445Thr showed destabilizing ΔΔG values, while p.Thr321Pro and p.Pro300Leu showed near‐neutral effects, suggesting a mechanism related to local conformational changes or altered inter‐subunit interactions rather than global protein destabilization (Table 2 and Figure 3). Some clinical observations from our cohort accord with these predictions: Patient 8, who exhibited a comparatively severe clinical phenotype, carried the p.Ile213Arg variant, which was associated with a high predicted protein instability score (ΔΔG = 2.65 kcal/mol). In contrast, Patients 6 and 7, both presenting with isolated ocular symptoms and sharing the same compound heterozygous genotype, showed a high instability score for p.Ile79Lys (ΔΔG = 2.61 kcal/mol) but a low score for p.Pro300Leu (ΔΔG = 0.19 kcal/mol). Furthermore, the p.Ile79Lys variant was previously reported as δI58K by Shen and colleagues in combination with a fast‐channel variant in Patient 4 of our cohort. Functional studies demonstrated reduced AChR abundance compared with wild‐type receptor, supporting the notion that structural alterations may contribute to impaired receptor expression [11]. However, while this patient presented with neonatal‐onset CMS and moderate disease severity, the same variant in combination with p.Pro300Leu was associated with a substantially milder phenotype in Patients 6 and 7. This observation further illustrates that the clinical consequences of individual CHRND variants not only depend on their intrinsic functional effects but also on the specific allelic context.

More broadly, pathogenic variants in AChR subunits are known to cause CMS due to several distinct molecular mechanisms. These include AChR deficiency due to impaired receptor assembly or trafficking, kinetic abnormalities such as fast‐channel syndromes with shortened channel openings or slow‐channel syndromes with prolonged channel openings, as well as receptor clustering at the neuromuscular junction [1]. The structural distribution of CHRND variants in our cohort suggests that multiple mechanisms may likewise contribute to disease pathogenesis. Variants located near inter‐subunit interfaces (Figure S3) may interfere with receptor assembly or stability, whereas variants located outside these regions are more likely to affect local folding or protein stability. These distinct molecular effects may result in different degrees of residual AChR function at the neuromuscular junction and thereby may contribute to the observed phenotypic variability. Nevertheless, structural and stability predictions did not consistently correlate with clinical severity. For example, Patient 4 carries both p.Ile79Lys and the strongly destabilizing p.Leu63Pro variant (ΔΔG = 3.48 kcal/mol), yet exhibits a relatively stable phenotype consisting of ocular manifestations together with proximal and facial muscle weakness. These findings highlight the limitations of current *in silico* approaches and suggest that additional factors influence disease manifestation. In the absence of dedicated functional studies, the proposed mechanistic interpretations remain hypothesis‐driven and future functional studies will be required to determine whether individual variants primarily affect receptor stability, assembly, trafficking, channel kinetics, or AChR‐rapsyn clustering.

Cognitive development was normal in the majority of patients, as expected for patients presenting with pathogenic *CHRND*‐variants. The presence of significant intellectual disability in Patient 8 therefore warrants particular caution in interpretation and may reflect other factors such as potential additional variants in genes associated with intellectual disability in light of parental consanguinity.

Treatment with pyridostigmine led to clinical improvement in most patients, with additional benefits seen in those treated with salbutamol/ephedrine or 3,4‐diaminopyridine. Patients with generalized weakness showed a positive but often incomplete response to pyridostigmine and frequently benefited from combination therapy. Ptosis improved in all treated patients, including those with exclusively ocular manifestations, whereas ophthalmoplegia showed little or no response. This finding is consistent with previous observations in ophthalmoparesis and ophthalmoplegia associated with other CMS subtypes [21]. Notably, Patient 9 received relatively high doses of pyridostigmine. The dose was titrated based on parent reports of functional capacity and frequency of inspiratory infections in combination with a high dependency on pyridostigmine. Clinical deterioration was documented when the dosage exceeded 15 mg/kg/day. However, overt cholinergic overmedication was not suspected. Supportive interventions such as gastrostomy and tracheostomy reflected disease severity and were required by the most severely affected individuals.

Comparison with previously reported *CHRND* cases confirms a consistent core phenotype characterized by ocular involvement (e.g., ptosis and ophthalmoparesis), with marked variability in disease severity (Table 3). Stratification by age at onset revealed that patients with prenatal or neonatal presentation shared a characteristic constellation of symptoms. Most cases presenting with prenatal or neonatal onset were characterized by muscular hypotonia, feeding and swallowing difficulties, respiratory involvement, and fatigable weakness. In an early report by Brownlow and colleagues, the index patient exhibited arthrogryposis multiplex congenita, indicating impaired neuromuscular transmission already during fetal development [6]. Severe phenotypes were associated with genotypes including one null allele. Lethal forms such as fetal akinesia sequence or multiple pterygium syndrome represent the extreme end of the spectrum [12, 15]. Biallelic missense variants were observed in milder, postnatal phenotypes, although variability remains substantial [18, 20]. Consistent with these earlier reports, our cohort recapitulates this phenotypic spectrum, ranging from severe neonatal disease requiring ventilatory and nutritional support to milder phenotypes with predominantly ocular involvement and preserved ambulation. Moreover, none of the patients in our cohort exhibited a prenatal lethal phenotype or features consistent with fetal akinesia sequence or lethal multiple pterygium syndrome. Moreover, electrophysiological findings in our cohort were consistent with previous reports, with a pathological decrement on repetitive nerve stimulation observed in patients with generalized weakness, whereas patients with purely ocular involvement showed normal or nondiagnostic studies.

Patient 2 of our cohort corresponds to the individual previously reported by Müller and colleagues in 2006 [8] and inclusion of this individual enables longitudinal follow‐up and extended clinical characterization beyond the initial report: compared with other neonatal‐onset patients, this case demonstrated marked improvement in neuromuscular symptoms during long‐term follow‐up, with sustained clinical stabilization and only stress‐ or infection‐triggered weakness, accompanied by persistent ocular manifestations under ongoing pyridostigmine therapy. A similarly favorable clinical course and response to pyridostigmine monotherapy were observed in Patient 3, who exhibited isolated ocular symptoms at last follow‐up. Two additional patients with long‐term follow‐up are Patients 4 (for additional 23 years after initial report) and 9 (for additional 1 years after initial report) previously reported by Shen and colleagues in 2008 and 2016, respectively [10, 11]. Patient 4 currently exhibits mild muscular combined with ocular symptoms, showing an improvement over time, albeit with add‐on therapy involving salbutamol/ephedrine. Patient 9 demonstrates a severe manifestation of the clinical spectrum seen in surviving patients, but despite treatment with pyridostigmine and 3,4 DAP, further deterioration has occurred, leading to the loss of her ability to sit unsupported and now requiring a wheelchair. Such clinical worsening under therapy has not been observed in any of the patients listed in Table 3 or in any other patient in our cohort.

Recent reports indicate that CMS represents an important differential diagnosis in generalized seronegative autoimmune myasthenia gravis (MG), including cases harboring *CHRND* variants with clinical onset in adolescence or even adulthood [21]. Based on the clinical presentation of our two cases with isolated ocular symptoms, *CHRND*‐related CMS should thus also be considered an important differential diagnosis in patients with a presumed diagnosis of ocular MG.

## 5 Conclusions

Recessive *CHRND*‐related CMS is characterized by a largely uniform clinical phenotype dominated by ocular involvement, with disease severity modulated by genotype and presumed residual acetylcholine receptor function. While genotypes including null alleles appear to be associated with an increased risk of severe, early‐onset generalized disease, biallelic missense variants show broader phenotypic variability and may result in either mild or more substantial neuromuscular involvement depending on their functional impact. Importantly, variability in clinical severity is well recognized in CMS, even among patients harboring identical or homozygous variants, suggesting that additional genetic, structural, or modifying factors contribute to the severity of disease manifestation. By expanding both the phenotypic and genotypic spectrum, including the identification of four novel missense variants (p.Ile213Arg, p.Pro300Leu, p.Thr321Pro, and p.Ile445Thr), this study provides further evidence for a continuum of disease severity in *CHRND*‐related CMS rather than discrete clinical subgroups. Our cohort's long‐term clinical follow‐up study, including previously reported patients, reveals a range of clinical outcomes, from marked improvement in neuromuscular symptoms with sustained stabilization to deterioration despite therapy. Together, these findings highlight the importance of integrating variant type, zygosity, clinical presentation, and residual receptor function for prognostication and therapeutic decision‐making in *CHRND*‐associated CMS.

## Author Contributions

**David Muhmann:** conceptualization, methodology, data curation, writing – original draft, writing – review and editing. **Angela Abicht:** data curation, writing – review and editing. **Andres Nascimento:** data curation, writing – review and editing. **Berta Estevez‐Arias:** data curation, writing – review and editing. **Silvia Cerezo Corredera:** data curation, writing – review and editing. **Damjan Osredkar:** data curation, writing – review and editing. **Adalet Elçin Yıldız:** data curation, writing – review and editing. **Maria Antonia Grimalt:** data curation, writing – review and editing. **Ulrike Schara‐Schmidt:** data curation, writing – review and editing. **Anja Troha Gergeli:** data curation, writing – review and editing. **Ana Vesperinas Castro:** data curation, writing – review and editing. **Göknur Haliloğlu:** data curation, writing – review and editing. **Elena Cortes Vicente:** data curation, writing – review and editing. **Johann Böhm:** data curation, writing – review and editing. **Adela Della Marina:** conceptualization, methodology, writing – original draft, writing – review and editing, data curation. **Andreas Roos:** conceptualization, methodology, data curation, writing – original draft, writing – review and editing. **Daniel Natera‐de Benito:** conceptualization, methodology, data curation, writing – original draft, writing – review and editing.

## Funding

D.M. acknowledges the support of the Junior Clinician Scientist Program from the University Medicine Essen, Duisburg‐Essen University. A.R. and A.D.M. acknowledge the support of the “Stiftung Universitätsmedizin Essen”.

## Ethics Statement

The study was approved by the local institutional review boards (University Medicine Essen: 19–9011‐BO; Hospital Sant Joan de Déu: PIC 147‐23) and conducted in accordance with the principles of the Declaration of Helsinki. Written informed consent to participate in the study, including genetic analyses, was obtained from all patients or their legal guardians.

## Consent

Written informed consent for the publication of clinical data was obtained from all patients or their legal guardians.

## Conflicts of Interest

The authors declare no conflicts of interest.

## Supporting information


**Figure S1:** Pedigrees of the present CHRND cohort.

**Figure S2:** Muscle ultrasonography findings in Patient 8: Muscle ultrasound studies show normal echogenicity and thickness in quadriceps femoris (a, Vastus lateralis, VL; Vastus intermedius, VI; rectus femoris, RF), hamstring muscles (b, Biceps femoris, BF; semitendinosus, ST; semimembranosus, SM), gracilis (c, Gr), soleus (d, Sol) flexor digitorum longus (FDL) and tibialis anterior (TA) (e), deltoid muscle (f, Del), sternocleidomastoid muscle (g, SCM) and increased echogenicity and mild atrophy of sartorius (c, St), medial and lateral heads of gastrocnemius (d, MH‐GC and LH‐GC) and paraspinal muscles (h, PS).

**Figure S3:** Structural localisation of CHRND missense variants mapped onto the human muscle acetylcholine receptor cryo‐EM structure (PDB: 9DMH): Variants are grouped according to their spatial relationship to inter‐subunit interfaces. Left panel: variants located outside direct inter‐subunit interfaces (p.Leu63Pro, p.Ile79Lys, p.Ile213Arg, p.Asp161Asn). Right panel: variants located in proximity to inter‐subunit interfaces (p.Val114Leu, p.Pro300Leu, p.Ile445Thr, p.Thr321Pro). The δ‐subunit is shown in magenta, neighboring subunits in distinct colors. Variant residues are highlighted in green. Of note: The p.Glu402Lys variant could not be reliably mapped onto the available cryo‐EM structure because the corresponding region was incompletely resolved in the structural model.

## Data Availability

The authors have nothing to report.

## References

[1] A. G. Engel , X. M. Shen , D. Selcen , and S. M. Sine , “Congenital Myasthenic Syndromes: Pathogenesis, Diagnosis, and Treatment,” Lancet Neurology 14, no. 4 (2015): 420–434, 10.1016/S1474-4422(14)70201-7. 25792100 PMC4520251

[2] K. Ohno , M. Ito , and B. Ohkawara , “Review of 40 Genes Causing Congenital Myasthenic Syndromes,” Journal of Human Genetics 70 (2025): 1–10, 10.1038/s10038-025-01355-9. 40533459

[3] D. Beeson , “Congenital Myasthenic Syndromes: Recent Advances,” Current Opinion in Neurology 29, no. 5 (2016): 565–571, 10.1097/WCO.0000000000000370. 27472506

[4] S. Ramdas and D. Beeson , “Congenital Myasthenic Syndromes: Where Do We Go From Here?,” Neuromuscular Disorders 31, no. 10 (2021): 943–954, 10.1016/j.nmd.2021.07.400. 34736634

[5] K. Ohno , B. Ohkawara , X. M. Shen , D. Selcen , and A. G. Engel , “Clinical and Pathologic Features of Congenital Myasthenic Syndromes Caused by 35 Genes‐A Comprehensive Review,” International Journal of Molecular Sciences 24, no. 4 (2023): 3730, 10.3390/ijms24043730. 36835142 PMC9961056

[6] S. Brownlow , R. Webster , R. Croxen , et al., “Acetylcholine Receptor Delta Subunit Mutations Underlie a Fast‐Channel Myasthenic Syndrome and Arthrogryposis Multiplex Congenita,” Journal of Clinical Investigation 108, no. 1 (2001): 125–130, 10.1172/JCI12935. 11435464 PMC209343

[7] J. R. Parr , M. J. Andrew , M. Finnis , D. Beeson , A. Vincent , and S. Jayawant , “How Common Is Childhood Myasthenia? The UK Incidence and Prevalence of Autoimmune and Congenital Myasthenia,” Archives of Disease in Childhood 99, no. 6 (2014): 539–542, 10.1136/archdischild-2013-304788. 24500997

[8] J. S. Müller , S. K. Baumeister , U. Schara , et al., “CHRND Mutation Causes a Congenital Myasthenic Syndrome by Impairing Co‐Clustering of the Acetylcholine Receptor With Rapsyn,” Brain 129, no. Pt 10 (2006): 2784–2793, 10.1093/brain/awl188. 16916845

[9] A. Della Marina , E. Wibbeler , A. Abicht , et al., “Long Term Follow‐Up on Pediatric Cases With Congenital Myasthenic Syndromes‐A Retrospective Single Centre Cohort Study,” Frontiers in Human Neuroscience 14 (2020): 560860, 10.3389/fnhum.2020.560860. 33364925 PMC7750519

[10] X. M. Shen , J. Brengman , D. Neubauer , S. M. Sine , and A. G. Engel , “Investigation of Congenital Myasthenia Reveals Functional Asymmetry of Invariant Acetylcholine Receptor (AChR) Cys‐Loop Aspartates,” Journal of Biological Chemistry 291, no. 7 (2016): 3291–3301, 10.1074/jbc.M115.683995. 26698174 PMC4751375

[11] X. M. Shen , T. Fukuda , K. Ohno , S. M. Sine , and A. G. Engel , “Congenital Myasthenia‐Related AChR Delta Subunit Mutation Interferes With Intersubunit Communication Essential for Channel Gating,” Journal of Clinical Investigation 118, no. 5 (2008): 1867–1876, 10.1172/JCI34527. 18398509 PMC2289798

[12] A. Michalk , S. Stricker , J. Becker , et al., “Acetylcholine Receptor Pathway Mutations Explain Various Fetal Akinesia Deformation Sequence Disorders,” American Journal of Human Genetics 82, no. 2 (2008): 464–476, 10.1016/j.ajhg.2007.11.006. 18252226 PMC2427255

[13] K. Polavarapu , B. Sunitha , A. Töpf , et al., “Clinical and Genetic Characterisation of a Large Indian Congenital Myasthenic Syndrome Cohort,” Brain 147, no. 1 (2024): 281–296, 10.1093/brain/awad315. 37721175 PMC10766255

[14] A. Troha Gergeli , D. Neubauer , T. Golli , et al., “Prevalence and Genetic Subtypes of Congenital Myasthenic Syndromes in the Pediatric Population of Slovenia,” European Journal of Paediatric Neurology 26 (2020): 34–38, 10.1016/j.ejpn.2020.02.002. 32070632

[15] C. Chen , J. Han , J. Xue , et al., “Case Report: Early Diagnosis of Lethal Multiple Pterygium Syndrome With Micrognathia: Two Novel Mutations in the *CHRND* Gene,” Frontiers in Genetics 14 (2023): 1005624, 10.3389/fgene.2023.1005624. 36733345 PMC9886669

[16] S. V. Tavtigian , S. M. Harrison , K. M. Boucher , and L. G. Biesecker , “Fitting a Naturally Scaled Point System to the ACMG/AMP Variant Classification Guidelines,” Human Mutation 41, no. 10 (2020): 1734–1737, 10.1002/humu.24088. 32720330 PMC8011844

[17] Y. Chen , H. Lu , N. Zhang , Z. Zhu , S. Wang , and M. Li , “PremPS: Predicting the Impact of Missense Mutations on Protein Stability,” PLoS Computational Biology 16, no. 12 (2020): e1008543, 10.1371/journal.pcbi.1008543. 33378330 PMC7802934

[18] X. M. Shen , K. Ohno , T. Fukudome , et al., “Congenital Myasthenic Syndrome Caused by Low‐Expressor Fast‐Channel AChR Delta Subunit Mutation,” Neurology 59, no. 12 (2002): 1881–1888, 10.1212/01.wnl.0000042422.87384.2f. 12499478

[19] H. Feng and H. Zhou , “New Compound Heterozygous Variants of the Cholinergic Receptor Nicotinic Delta Subunit Gene in a Chinese Male With Congenital Myasthenic Syndrome: A Case Report,” Medicine (Baltimore) 96, no. 51 (2017): e8981, 10.1097/MD.0000000000008981. 29390429 PMC5758131

[20] C. Bonanno , C. Rodolico , A. Töpf , et al., “Severe Congenital Myasthenic Syndrome Associated With Novel Biallelic Mutation of the CHRND Gene,” Neuromuscular Disorders 30, no. 4 (2020): 336–339, 10.1016/j.nmd.2020.02.012. 32360402

[21] J. Theuriet , M. Masingue , A. Behin , et al., “Congenital Myasthenic Syndromes in Adults: Clinical Features, Diagnosis and Long‐Term Prognosis,” Brain 147, no. 11 (2024): 3849–3862, 10.1093/brain/awae124. 38696726 PMC11531845

