# Supplementary material for: Clinical Variability and Genotype‐Driven Outcomes in CHRND‐Related Congenital Myasthenic Syndrome
Source: Eur J Neurol. 2026 Aug 27;33(9):e70742. doi: 10.1111/ene.70742 (PMC13520113; doi:10.1111/ene.70742)
Supplement: Supplementary file 1 [file ENE-33-e70742-s002.pdf]

Variants outside inter-subunit interfaces

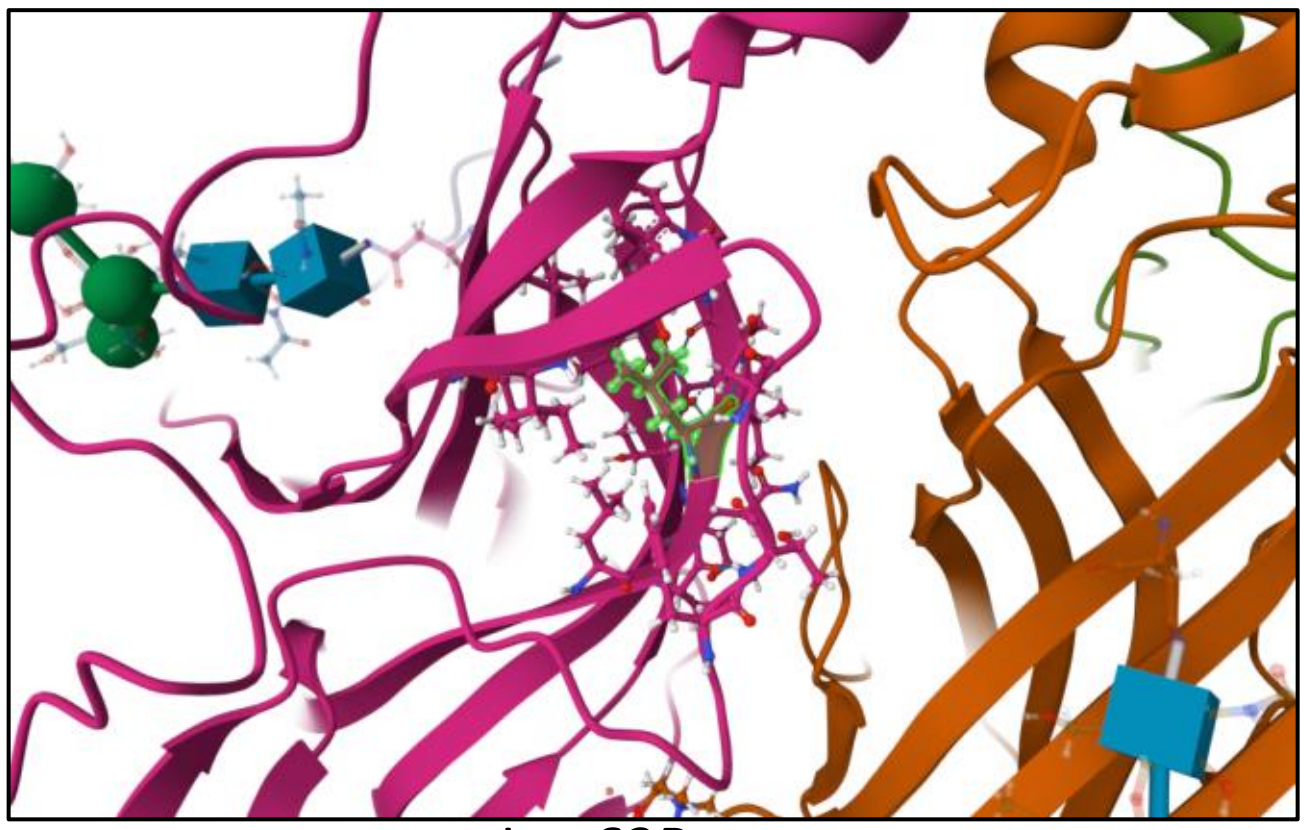

p.Leu63Pro

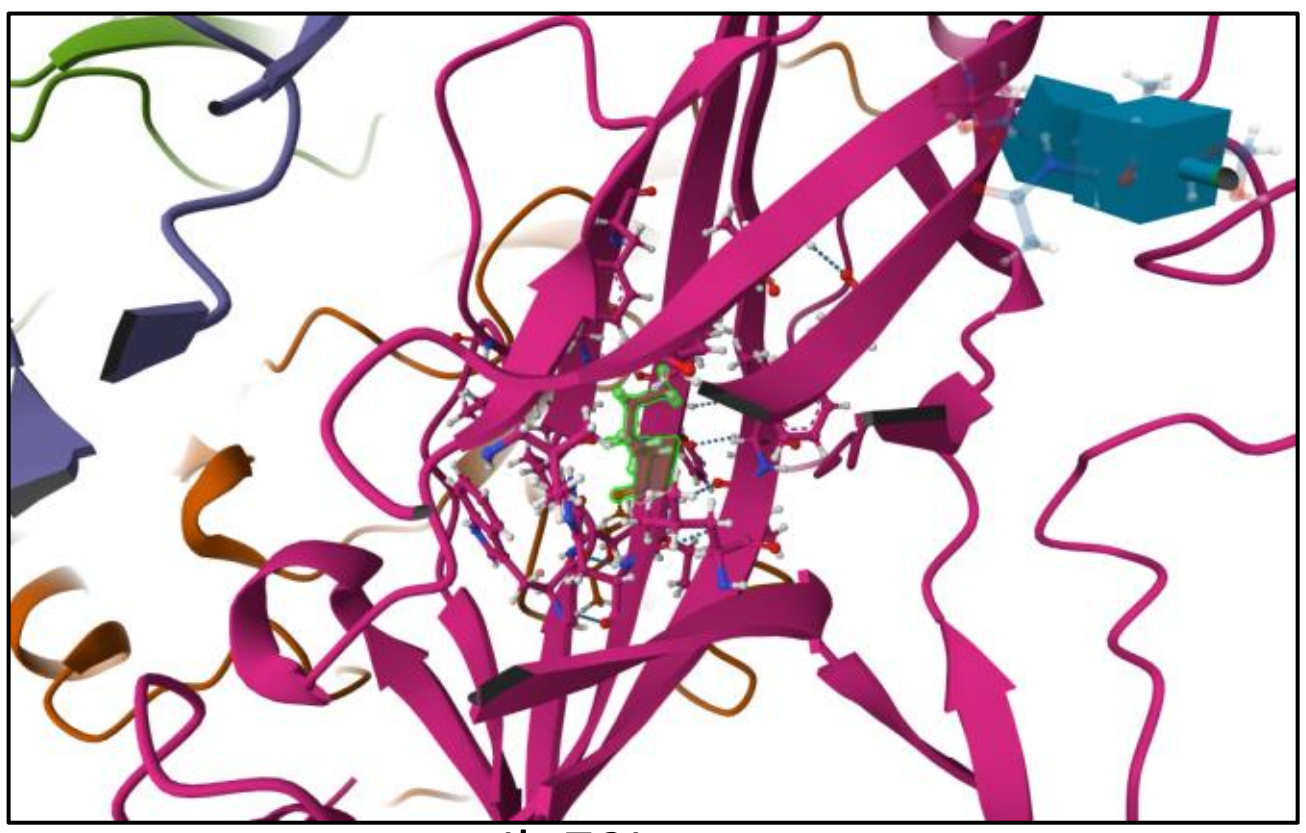

p.Ile79Lys

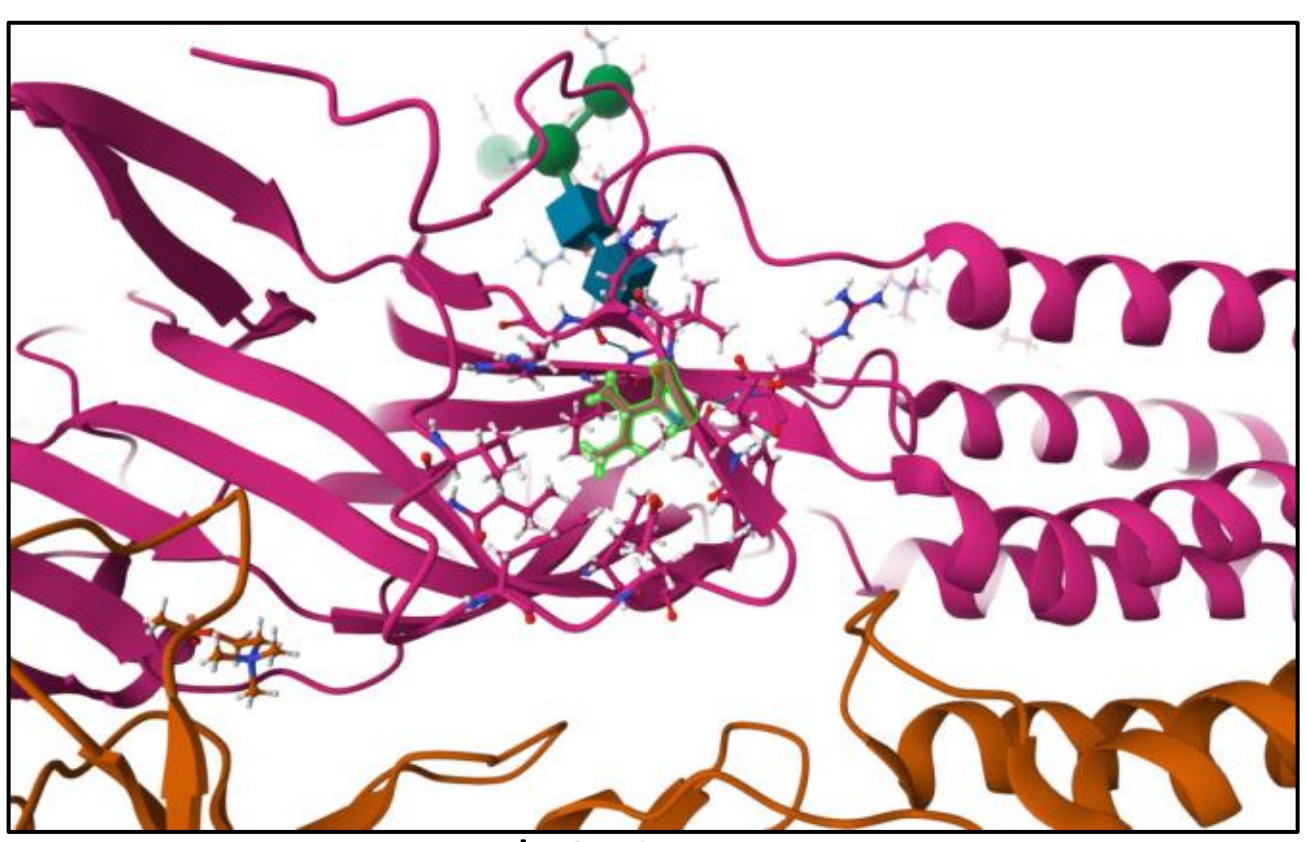

p.Ile213Arg

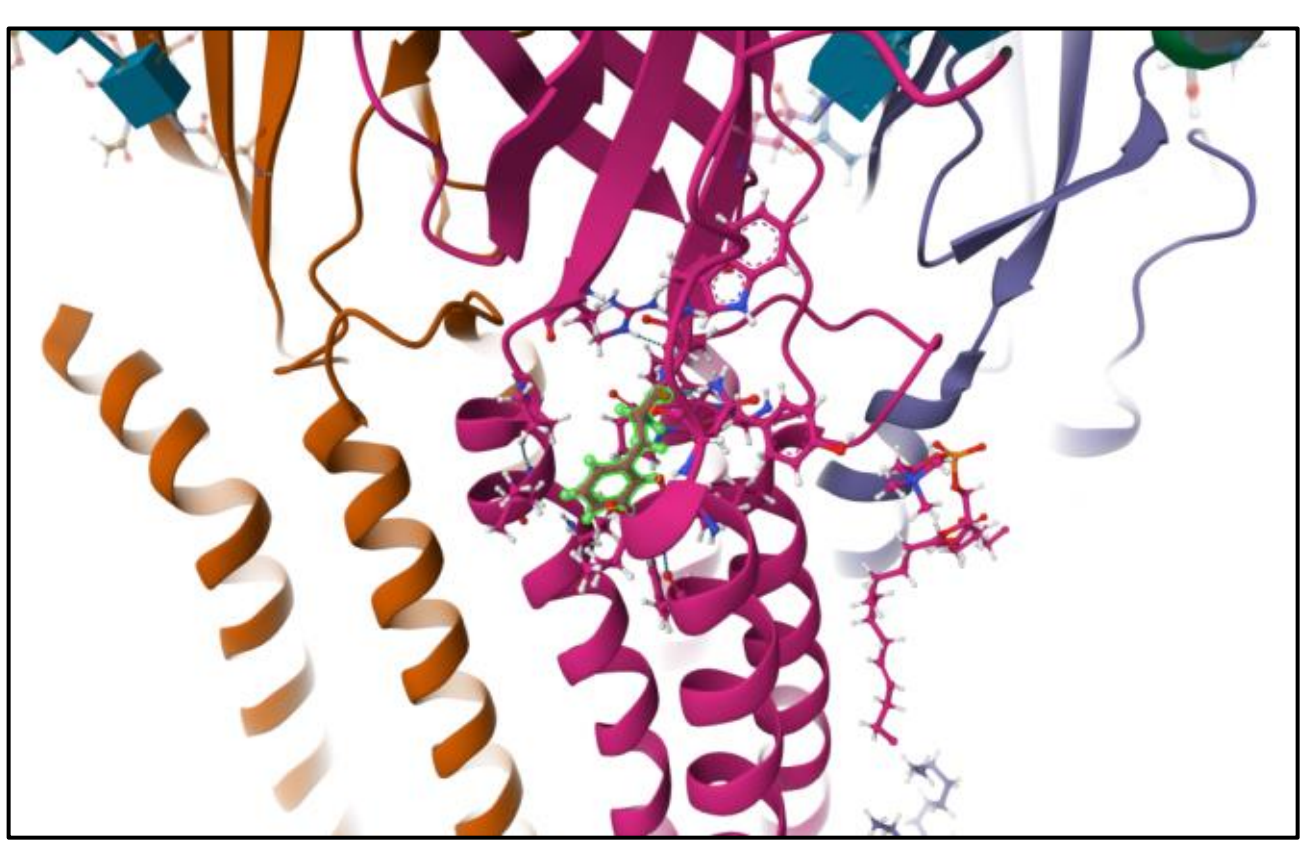

p.Asp161Asn

Variants near inter-subunit interfaces

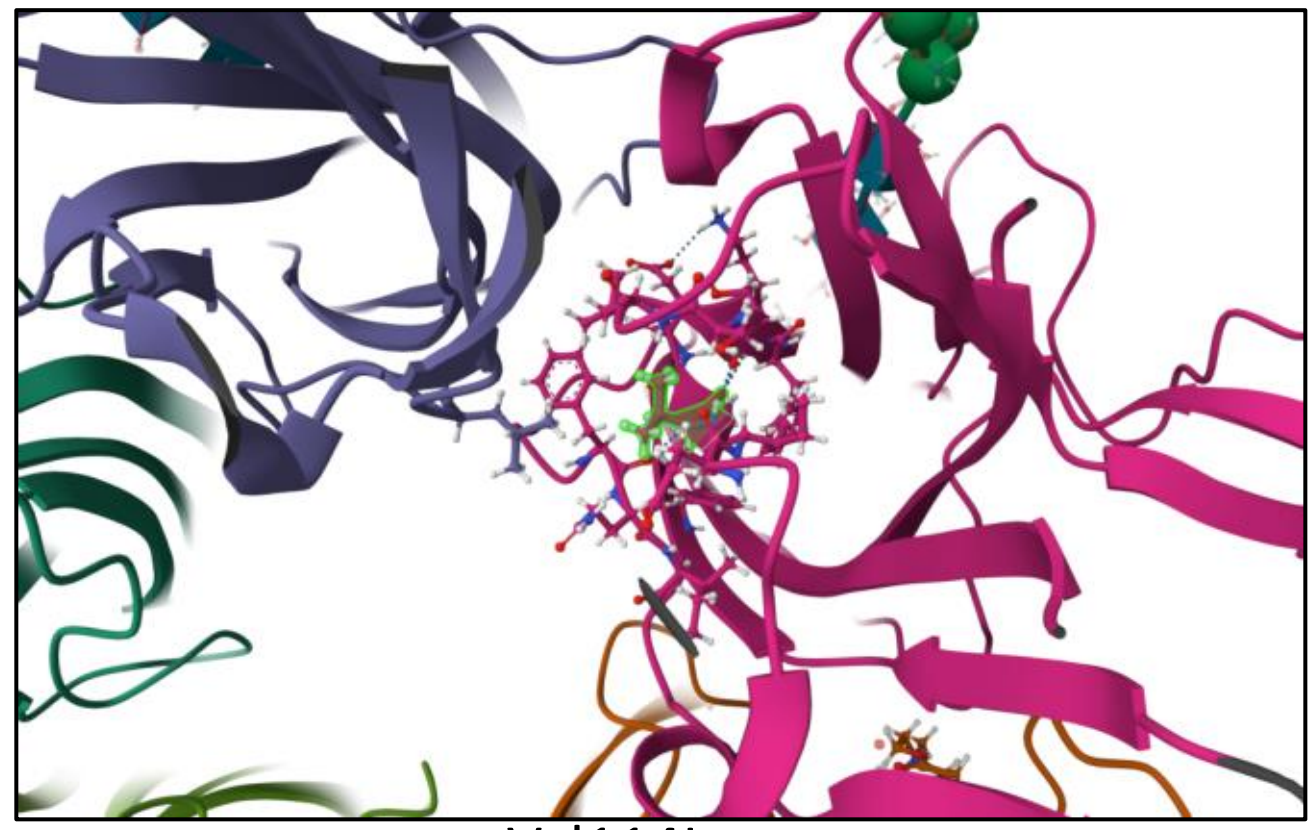

p.Val114Leu

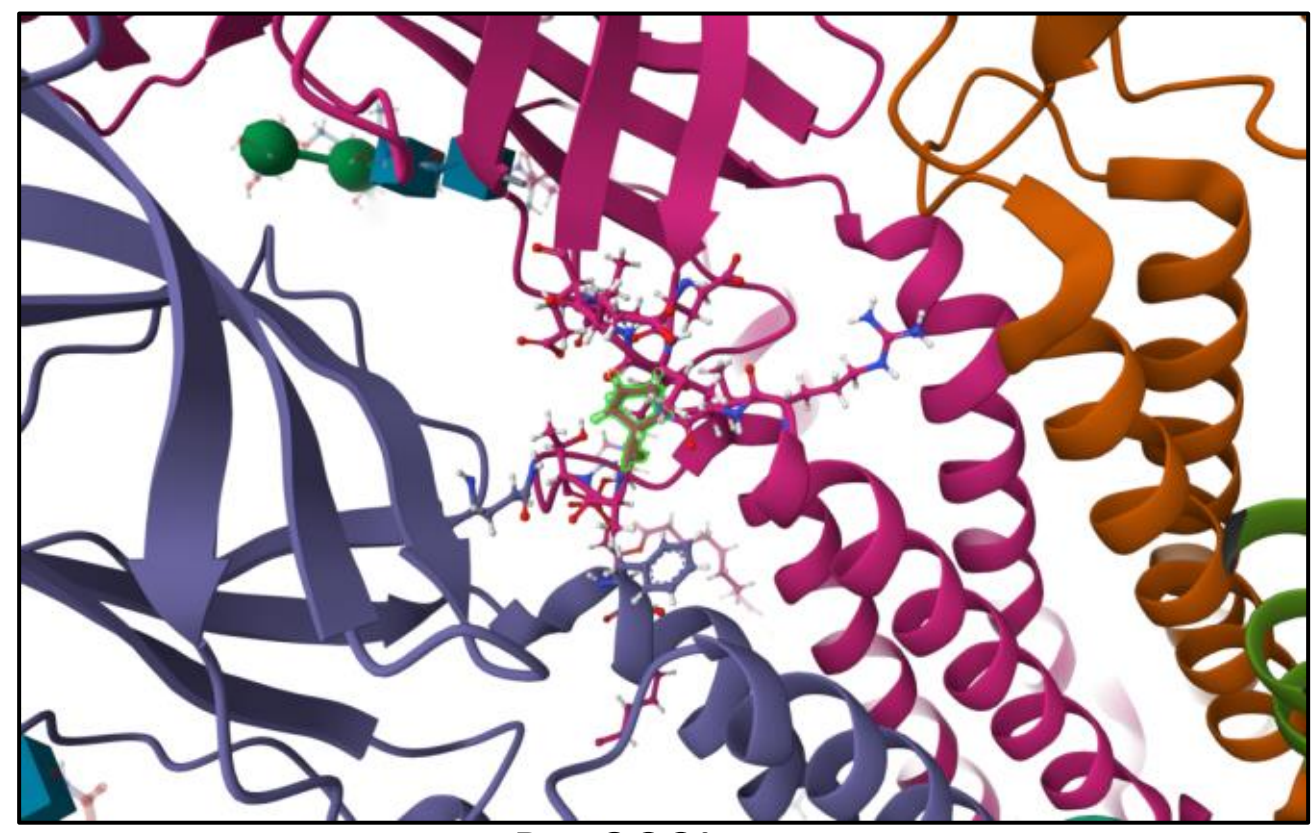

p.Pro300Leu

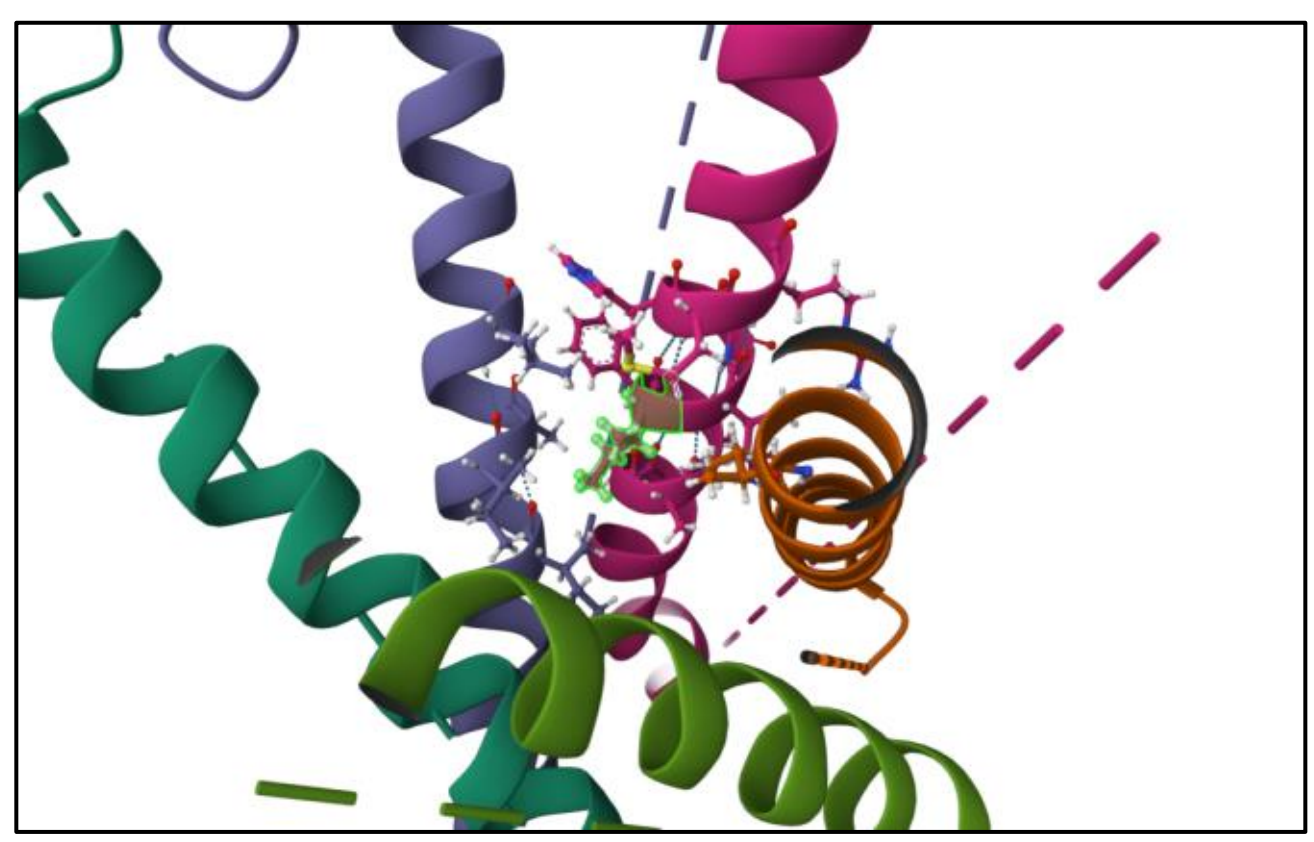

p.Ile445Thr

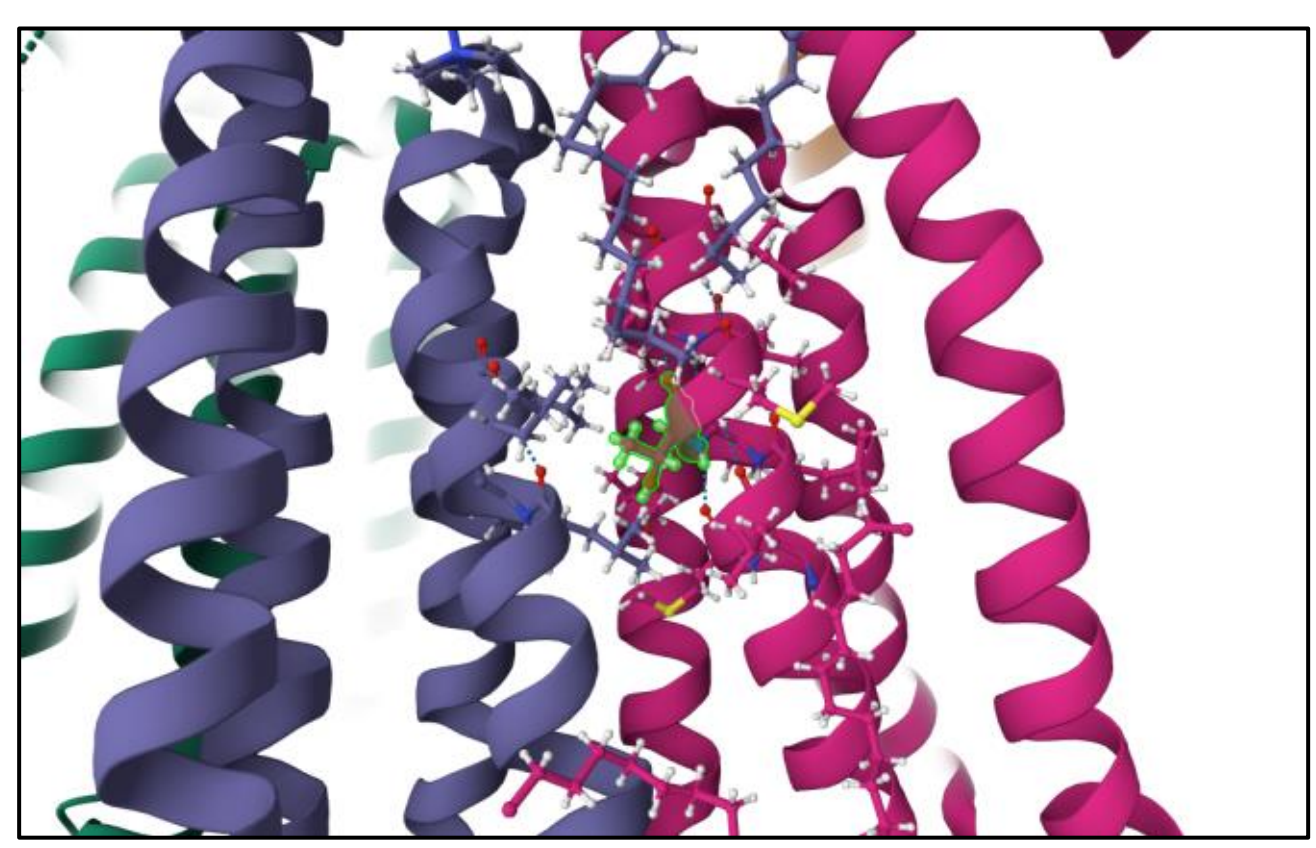

p.Thr321Pro
